# Supplementary material for: Dynamic action of the Sec machinery during initiation, protein translocation and termination
Source: eLife. 2018 Jun 7;7:e35112. doi: 10.7554/eLife.35112 (PMC6021171; doi:10.7554/eLife.35112)
Supplement: Transparent reporting form [file elife-35112-transrepform.docx]

***eLife’s* transparent reporting form**

We encourage authors to provide detailed information *within their submission* to facilitate the interpretation and replication of experiments. Authors can upload supporting documentation to indicate the use of appropriate reporting guidelines for health-related research (see [EQUATOR Network](http://www.equator-network.org/%20)), life science research (see the [BioSharing Information Resource](https://biosharing.org/)), or the [ARRIVE guidelines](http://www.plosbiology.org/article/info:doi/10.1371/journal.pbio.1000412) for reporting work involving animal research. Where applicable, authors should refer to any relevant reporting standards documents in this form.

If you have any questions, please consult our Journal Policies and/or contact us: [editorial@elifesciences.org](mailto:editorial@elifesciences.org).

**Sample-size estimation**

- You should state whether an appropriate sample size was computed when the study was being designed
- You should state the statistical method of sample size computation and any required assumptions
- If no explicit power analysis was used, you should describe how you decided what sample (replicate) size (number) to use

Please outline where this information can be found within the submission (e.g., sections or figure legends), or explain why this information doesn’t apply to your submission:

For single molecule experiments sample size is determined by the number of events, e.g. number of labelled molecules passing through confocal volume and giving off fluorescence, and it is stated in the in figure legends of Figure 2E,G and Figure 4a. These numbers apply to all other statistical analyses based on either confocal or TIRF technique.

**Replicates**

- You should report how often each experiment was performed
- You should include a definition of biological versus technical replication
- The data obtained should be provided and sufficient information should be provided to indicate the number of independent biological and/or technical replicates
- If you encountered any outliers, you should describe how these were handled
- Criteria for exclusion/inclusion of data should be clearly stated
- High-throughput sequence data should be uploaded before submission, with a private link for reviewers provided (these are available from both GEO and ArrayExpress)

Please outline where this information can be found within the submission (e.g., sections or figure legends), or explain why this information doesn’t apply to your submission:

- Biological and technical replications are appropriately described in text.
- No outliers were excluded from data analysis

**Statistical reporting**

- Statistical analysis methods should be described and justified
- Raw data should be presented in figures whenever informative to do so (typically when N per group is less than 10)
- For each experiment, you should identify the statistical tests used, exact values of N, definitions of center, methods of multiple test correction, and dispersion and precision measures (e.g., mean, median, SD, SEM, confidence intervals; and, for the major substantive results, a measure of effect size (e.g., Pearson's r, Cohen's d)
- Report exact p-values wherever possible alongside the summary statistics and 95% confidence intervals. These should be reported for all key questions and not only when the p-value is less than 0.05.

Please outline where this information can be found within the submission (e.g., sections or figure legends), or explain why this information doesn’t apply to your submission:

- least squares methods were used for fitting physical models and extracting parameters as described and referenced in methods
- Ordinary least squares linear regression was used to estimate the translocation rate in Figure 5A using all data
- Applied statistical methods and used variables are described in section methods on pages 17-21.

(For large datasets, or papers with a very large number of statistical tests, you may upload a single table file with tests, Ns, etc., with reference to sections in the manuscript.)

**Group allocation**

- Indicate how samples were allocated into experimental groups (in the case of clinical studies, please specify allocation to treatment method); if randomization was used, please also state if restricted randomization was applied
- Indicate if masking was used during group allocation, data collection and/or data analysis

Please outline where this information can be found within the submission (e.g., sections or figure legends), or explain why this information doesn’t apply to your submission:

No groups allocated in this work

**Additional data files (“source data”)**

- We encourage you to upload relevant additional data files, such as numerical data that are represented as a graph in a figure, or as a summary table
- Where provided, these should be in the most useful format, and they can be uploaded as “Source data” files linked to a main figure or table
- Include model definition files including the full list of parameters used
- Include code used for data analysis (e.g., R, MatLab)
- Avoid stating that data files are “available upon request”

Please indicate the figures or tables for which source data files have been provided:

In this manuscript, we used open source software, as noted in the methods section. For processing of TIRF data, we used iSMS software (Preus et al.,2015).

Fluorescence bursts were analysed by customized python programming scripts based on the open source toolkit for analysis of freely-diffusing single-molecule FRETbursts (Ingargiola et al.,2016b). The most important addition to FRETbursts is global fitting of RASP kinetics. This code is available at following link:

<https://drive.google.com/file/d/1Z2YOWDy-4OBkTvYOReTV1ADys2wh3z2d/view?usp=sharing>
